# Transmission and prevalence of drug-resistant tuberculosis in a Brazilian setting under a directly observed therapy short-course strategy

**DOI:** 10.1590/0037-8682-0404-2019

**Published:** 2020-06-22

**Authors:** Fábio Oliveira Latrilha, Vera Simonsen, Juliana Maira Watanabe Pinhata, Angela Pires Brandão, Vera Maria Neder Galesi, Eliseu Alves Waldman, Lucilaine Ferrazoli

**Affiliations:** 1 Instituto Adolfo Lutz, Centro de Bacteriologia, Núcleo de Tuberculose e Micobacterioses, São Paulo, SP, Brasil.; 2 Fundação Oswaldo Cruz, Instituto Oswaldo Cruz, Rio de Janeiro, RJ, Brasil.; 3 Secretaria de Saúde do Estado de São Paulo, Centro de Vigilância Epidemiológica “Prof. Alexandre Vranjac”, Divisão de Controle da Tuberculose, São Paulo, SP, Brasil.; 4 Universidade de São Paulo, Faculdade de Saúde Pública, São Paulo, SP, Brasil.

**Keywords:** Brazil, Directly observed treatment, Drug resistance, RFLP, Transmission, Tuberculosis

## Abstract

**INTRODUCTION:**

We aimed to estimate the prevalence and transmission of drug-resistant tuberculosis in a high-burden Brazilian setting under directly observed therapy short-course strategy.

**METHODS:**

Isolates of culture-confirmed pulmonary tuberculosis patients from Guarulhos, Brazil, diagnosed in October 2007-2011 were subjected to drug susceptibility and IS*6110*-restriction fragment length polymorphism testing.

**RESULTS:**

The overall resistance prevalence was 11.5% and the multi-drug resistance rate was 4.2%. Twenty-six (43.3%) of 60 drug-resistant isolates were clustered. Epidemiological relationships were identified in 11 (42.3%) patients; 30.8% of the cases were transmitted in households.

**CONCLUSIONS:**

Drug-resistant tuberculosis was relatively low and transmitted in households and the community.

Brazil remains among the 20 countries worldwide with the highest number of tuberculosis (TB) cases^1^. The World Health Organization has recommended the use of the directly observed therapy short-course (DOTS) strategy to increase cure rates, reduce losses to follow-up, prevent the occurrence of new cases of drug resistance, and reduce the transmission of *Mycobacterium tuberculosis* complex (MTBC) strains^2^.

In 1996, the National Tuberculosis Program of Brazil (NTP) implemented the Emergency Action Plan for TB control. This plan initially prioritized 206 municipalities and was later expanded to 230 municipalities. Since then, the NTP has recommended the implementation of the DOTS strategy, mainly in the priority municipalities^3^.

Implementation of the DOTS strategy started in 1998 in Sao Paulo state. Between 2004 and 2011, the Surveillance Center of Sao Paulo state, in partnership with the United States Agency for International Development, intensified the DOTS strategy in Sao Paulo, Carapicuiba, and Guarulhos.

The emergence and spread of drug-resistant MTBC strains have threatened TB control globally^1^. Restriction fragment length polymorphism (RFLP) and spoligotyping have been applied in the study of TB transmission to improve public health surveillance^4^ ^,^ ^5^ ^,^ ^6^.

This study aimed to estimate the prevalence of resistance to first-line anti-TB drugs as well as the transmission of drug-resistant MTBC strains in a high-burden Brazilian setting using a DOTS strategy.

This cross-sectional study was performed in Guarulhos, a city with >1.3 million inhabitants^7^ and a TB incidence of 31/100,000 inhabitants. Guarulhos has 67 primary health care units and one public health laboratory (PHLG) that performs smear microscopy and culturing. Mycobacteria cultures are systematically referred to the Adolfo Lutz Institute (IAL) for further identification, drug susceptibility testing (DST), and molecular typing. In Brazil culture and DST for TB are performed mainly for groups at high risk of TB resistance^8^.

For this study, the reference population consisted of all pulmonary TB (PTB) patients of both sexes aged ≥ 15 years residing, diagnosed, and treated in Guarulhos between October 2007 and October 2011. Patients meeting these criteria, whose MTBC isolates were subjected to DST at the IAL, were enrolled in the study. Patients who received 24 supervised doses in the intensive phase and 48 doses in the second phase were classified as having completed the DOTS^8^. 

The patients’ sociodemographic and clinical information was collected from the TB notification system of Sao Paulo state (TBWEB). The laboratory results of smear microscopy and culturing were collected at the PHLG, while DST results were collected at the IAL. 

MTBC isolates referred to the IAL by the PHLG underwent first-line DST (isoniazid [INH], streptomycin [SM], rifampin [RIF], ethambutol [EMB]) using a BACTEC Mycobacterial Growth Indicator Tube™ 960 system (Becton & Dickson, Maryland, USA). Resistance to pyrazinamide was also detected using the pyrazinamidase test^4^.

Since 2007, the IAL has performed RFLP genotyping of all drug-resistant MTBC isolates received from approximately 80 laboratories in Sao Paulo state. RFLP was performed as previously described^4^ and the patterns were analyzed by Bionumerics software (Applied Maths, Kortrijk, Belgium). Patients whose isolates presented identical numbers of DNA bands of the same molecular weight or one band difference were considered to be in a cluster, indicative of recent transmission. Isolates showing fewer than six IS*6110* copies without an epidemiological link were excluded. RFLP patterns identified in this study were compared to all patterns of the Sao Paulo state database to include patients residing in Guarulhos but treated outside that city during the same study period.

The statistical analysis was performed using Epi-Info version 3.5 software (Centers for Disease Control and Prevention). Pearson’s chi-square test with Yates correction or Fisher’s exact test were used to examine categorical variables. Means were compared by the Kruskal Wallis test. *P* values ≤ .05 were considered statistically significant.

A total of 1,538 TB cases were identified in Guarulhos between October 2007 and October 2011. Of them, 1,280 (83.2%) were PTB, 231 (15.0%) were extrapulmonary TB (EPTB), 22 (1.5%) were PTB associated with EPTB (PTB+EPTB), and 5 (0.3%) were disseminated TB. Among the 1,302 PTB and PTB+EPTB patients (reference population), 476 (36.6%) were culture-positive for MTBC. Of them, 407 (85.5%) underwent DST (study population), which represent 31.3% of all PTB patients reported in the study period. Among these 407 PTB patients, 349 (85.7%) were new cases and the remaining 58 (14.3%) were previously treated for TB (Figure 1). 

**FIGURE 1:**
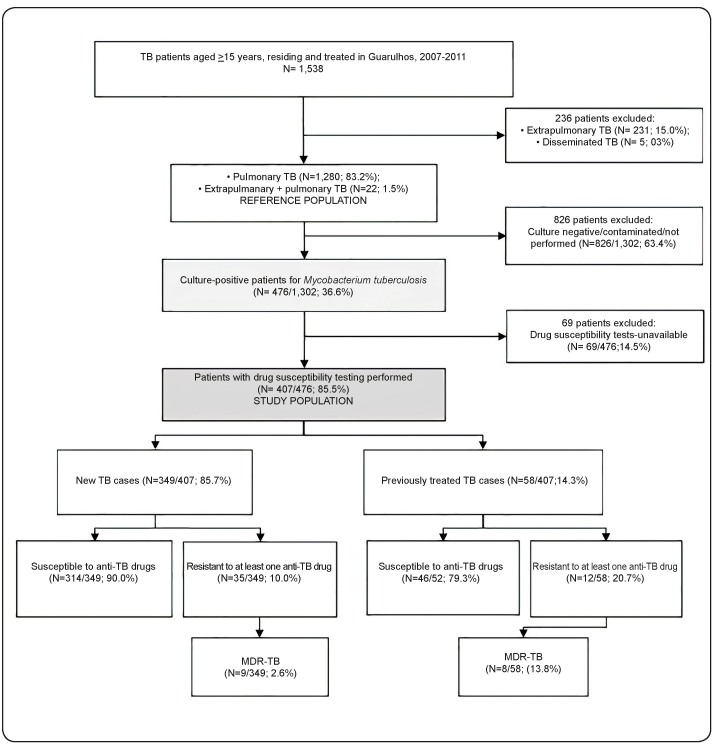
Study population selection, tuberculosis form, treatment, and drug susceptibility results of patients from Guarulhos, October 2007-2011. **MDR:** multidrug resistant; **TB:** tuberculosis. Anti-TB drugs tested included isoniazid, rifampicin, pyrazinamide, streptomycin, and ethambutol.

The reference and study populations showed similar characteristics regarding sex, years of education, and HIV coinfection. However, the study population was younger (mean 34.5 ± 13.7 years vs. 37.4 ± 14.7 years; *p* < .001), with higher frequencies of inmates (26.5% vs. 9.8%; *p* < .001) and retreatment cases (14.2% vs. 7.8%; *p* < .001).

Of the 407 patients, 335 (82.3%) started DOTS, but only 54.1% (220/407) completed the course of at least 72 supervised doses.

The sociodemographic and clinical characteristics of the study population stratified by new and previously treated patients are shown in Table 1. Regarding the DST pattern, in the study population, 47 of 407 (11.5%) patients showed resistance to at least one of the first-line anti-TB drug; of them, 17 (4.2%) were multidrug-resistant TB (MDR-TB) and 15 (3.7%) were INH-monoresistant cases (Table 1).

**TABLE 1:** Characteristics of the 407 pulmonary tuberculosis patients according to treatment history, Guarulhos, October 2007-2011.

Characteristic	Total (407)		New cases ( 349)		Previous TB (58)		*P*
	N	%	n	%	n	*%*	
**Sex**							
Male	302	74.2	256	73.4	46	79.3	.424
Female	105	25.8	93	26.6	12	20.7	
**Age (yrs)**							
15-19	34	8.3	32	9.2	2	3.4	.152
20-39	247	60.7	214	61.3	33	56.9	
>40	126	31.0	103	29.5	23	39.7	
**Mean age (yrs)**	34.6 ±13.7 13.7	-	34.2 ± 14	-	37.2 ± 11.4	-	.003
**Education (yrs)**							
0-3	46	11.3	42	12.0	4	6.9	.371
4-11	257	63.1	226	64.8	31	53.4	
≥12	13	3.2	10	2.9	3	5.2	
Unknown	91	22.4	71	20.3	20	34.5	
**History of imprisonment**							
Yes	108	26.5	96	27.5	12	20.7	.353
No	299	73.5	253	72.5	46	79.3	
**HIV status**							
Positive	27	6.7	21	6.0	6	10.3	.348
Negative	351	86.2	303	86.8	48	82.8	
Unknown	29	7.1	25	7.2	4	6.9	
**Treatment outcome**							
Cured/completed	346	85.0	303	86.8	43	74.2	.081
Lost to follow-up	46	11.3	35	10.0	11	19.0	
Died of TB	6	1.5	4	1.2	2	3.4	
Others	9	2.2	7	2.0	2	3.4	
**Treatment type**							
DOTS	220	54.0	190	54.4	30	51.7	.808
SAT	187	46.0	159	45.6	28	48.3	
**Sputum smear**							
Positive	358	88.0	307	88.0	52	89.7	.862
Negative	35	8.6	29	8.3	6	10.3	
Not done	14	3.4	13	3.7	-	-	
**Drug resistance**							
Susceptible	360	88.4	314	90.0	46	79.3	<.001
Multidrug-resistant	17	4.2	9	2.6	8	13.8	
Mono/polyresistant	30	7.4	26	7.4	4	6.9	
INH	15	3.7	13	3.7	2	3.4	
SM	11	2.7	10	2.9	1	1.7	
INH+SM	4	1.0	3	0.8	1	1.7	

**DOTS:** directly observed therapy short-course; **HIV:** human immunodeficiency virus; **INH:** isoniazid; **SAT:** self-administered treatment; **SM:** streptomycin; **TB:** tuberculosis.

Among the new cases, the prevalence of resistance to at least one drug was 10.0% (35/349), while that of monoresistance to INH was 3.7% (13/349) and that of MDR-TB was 2.6% (9/349). Regarding previously treated patients, the prevalence of resistance was 20.7% (12/58) to at least one drug, 3.4% (2/58) to INH alone, and 13.8% (8/58) to INH and RIF (MDR-TB). Compared to the new cases, previously treated patients were significantly more likely to be MDR (*p* < .001).

RFLP typing was performed on 43 of the 47 drug-resistant isolates because four did not grow in subculture. RFLP patterns were analyzed in two steps. First, the RFLP patterns of the 43 MTBC isolates from the study population were compared. Then, they were compared to all patterns in the RFLP database of drug-resistant MTBC isolates of Sao Paulo state. In this second step, 20 additional patients residing in Guarulhos but treated in Sao Paulo city were included. Analysis of the RFLP patterns of these 63 patients showed that the number of IS*6110* copies ranged from one to 19. Only seven isolates exhibited fewer than five copies. Two of them presenting one and three copies were classified as unique patterns. Of the remaining five isolates, each displaying four copies, two isolates had an epidemiological link and were kept in the study. The other three isolates were excluded from the analysis, resulting in 60 MTBC isolates. 

As shown in Figure 2, 26 (43.3%) of the 60 TB cases belonged to five clusters (SP5, SP5W, SP1a, SP12b, and 4a). The SP5 cluster was the most prevalent, comprising 16 (61.5%) patients, of whom 14 (87.5%) had MDR-TB and two were INH-resistant. In one case, the SP5 pattern was mixed with another RFLP pattern (SP5 mix), probably due to laboratory contamination or mixed infection. Of the four remaining clusters, one comprised four patients and the other three comprised two patients each.

**FIGURE 2:**
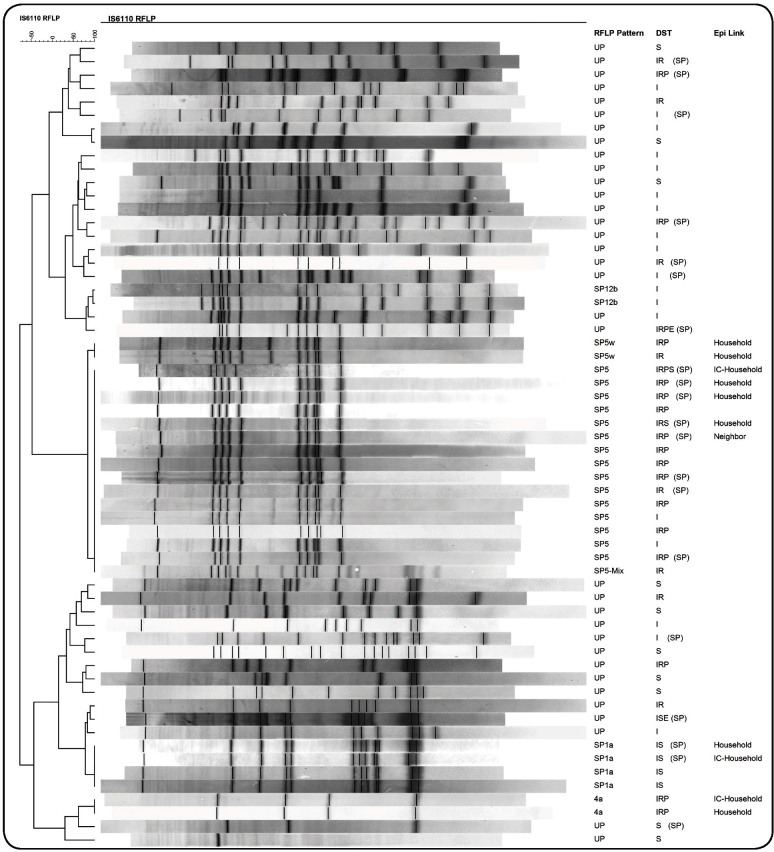
Restriction fragment length polymorphism patterns (RFLP) of 60 tuberculosis patients living in the municipality of Guarulhos, of whom 40 were treated in Guarulhos and 20 were treated in the municipality of Sao Paulo, October 2007-2011.

DST: drug susceptibility testing; E: ethambutol; Epi Link: epidemiological link; I: isoniazid; IC: index case; P: pyrazinamide; R: rifampicin; S: streptomycin; SP: patients treated in Sao Paulo City; UP: unique pattern. 

The sociodemographic and clinical characteristics of the clustered and unclustered patients were compared. Clustered patients presented similar frequencies regarding sex (*p* = .738), age (range, 15-89 years, *p* = .957), years of education (*p* = .092), history of imprisonment (*p* = .677), HIV status (*p* = .599), treatment history (*p* = .468), treatment outcome (*p* = .727), and sputum smear microscopy results (*p* = .969). Compared to the unclustered patients, clustered patients were significantly more likely to be MDR (69.2% vs 26.5%; *p* = .002) and were under DOTS (65.4% vs 29.4%; *p* = .011). 

Among the 26 patients belonging to clusters, epidemiological links were identified in 8 (30.8%). All of these patients had household or neighbor contact with TB. 

 The drug-resistant TB prevalence found in this study is similar to those of previous studies performed in different states of Brazil. According to these studies, the overall prevalence of INH monoresistance and MDR for new cases was 3.3-4.9% and 1.0-2.2%, respectively^4^ ^-^ ^9^. The MDR-TB prevalence of 2.6% among new cases found in Guarulhos is higher than the 1.4% found in Brazil during the II National Anti-Tuberculosis Drug Resistance Survey conducted in 2006-2007^10^ and lower than the global prevalence (3.5%) of MDR-TB/RIF-resistant TB reported in 2014^11^. In contrast with the drug resistance prevalence in new TB patients, the rates among retreatment cases had greater variability according to these studies. The rates of INH resistance and MDR were 4.3-17.3% and 12.0-16.7%, respectively^4^ ^-^ ^9^.

 Almost half of INH-resistant or MDR-TB cases in our study occurred due to recent transmission. Previous studies highlighted the impact of DOTS on reducing transmission and incidence of drug-susceptible and -resistant TB in places with moderate and high rates of drug resistance. However, a study conducted in Taiwan demonstrated that, despite the reduction in drug-resistant TB rates, the prevalence of primary drug resistance remained stable during the 7-year study period^12^. In our study, trends in resistance or transmission prevalence were not assessed. However, 69.4% of INH-resistant or MDR-TB patients had no previous history of anti-TB treatment, which may suggest that these patients acquired resistant TB due to recent transmission. In this study, the findings of primary drug resistance were corroborated by our molecular typing results. We found that 48.0% of the INH-resistant or MDR isolates belonged to cluster patterns, meaning that drug-resistant TB was recently transmitted among these patients.

The rate of monoresistance to SM was lower (2.7%) than that to INH (3.7%). All 11 SM monoresistant patients showed a unique RFLP pattern indicating the potential reactivation of a previous infection, since SM is not used to treat new cases. Similar findings were reported by Telles et al.^4^. 

Epidemiological links were found for almost half of the clustered patients. Considering that there were three index cases, the transmission rate among cases with epidemiological links was 30.8%. Except for one case (neighbor), all contacts were relatives, *i.e.,* household transmission of drug-resistant isolates occurred frequently in this setting. The household transmission rate was similar to those found in studies performed in Brazil and elsewhere^6^ ^-^ ^13^ and could be explained by the fact that drug-resistant TB has a prolonged infectious period while the patient was under treatment. 

The lack of epidemiological links among the majority of the drug-resistant cases (69.2%) suggests ongoing transmission in the community. Studies conducted since the early 1990s using molecular typing have identified a significant proportion of transmission of drug-resistant strains in hospitals, prisons, or in the community^4^ ^,^ ^5^ ^,^ ^6^. 

During the study period, Guarulhos improved the DOTS coverage: 82% of the patients started the DOTS course and 54% completed 72 supervised doses compared with 1998-2004, when the DOTS coverage was lower (40%)^14^. This proportion is higher than the national coverage of 38.3% for patients aged ≥ 18 years reported by Reis-Santos and colleagues in 2011^15^. 

There are several methodological limitations to our study. First, it was a retrospective study based on secondary data. Second, no formal evaluation was performed to ensure the accuracy of the DOTS information available on the TBWEB system. A comparative analysis between the study and reference populations showed no difference between them in the main clinicodemographic characteristics. However, there were higher numbers of inmates and retreatment patients in the study population, possibly because of intensification of culture and DST for people at risk of developing drug resistance. Finally, we did not genotype the susceptible MTBC isolates. 

In conclusion, the prevalence of drug-resistant TB in Guarulhos was relatively low, and the disease was transmitted via household and community contacts. DOTS is an important strategy for reducing loss to follow-up rates and increasing cure rates, although additional efforts such as the rapid molecular diagnosis of drug resistance and improvements in contact investigation are needed to reduce the transmission of drug-resistant TB.
